# Therapy-refractory Panton Valentine Leukocidin-positive community-acquired methicillin-sensitive Staphylococcus aureus sepsis with progressive metastatic soft tissue infection: a case report

**DOI:** 10.1186/1752-1947-1-165

**Published:** 2007-12-03

**Authors:** Joerg C Schefold, Fabrizio Esposito, Christian Storm, Dagmar Heuck, Anne Krüger, Achim Jörres, Wolfgang Witte, Dietrich Hasper

**Affiliations:** 1 Department of Nephrology and Medical Intensive Care, Charité University Medicine, Campus Virchow Clinic, Berlin, Germany; 2 Robert Koch Institut, National Staphylococcal Reference Centre, Wernigerode Branch, Wernigerode, Germany

## Abstract

We report a case of fulminant multiple organ failure including the *Acute Respiratory Distress Syndrome *(ARDS), haemodynamic, and renal failure due to community-acquired *methicillin-sensitive *Panton Valentine Leukocidin (PVL) positive *spa*-type 284 (ST121) *Staphylococcus aureus *septic shock. The patient's first clinical symptom was necrotizing pneumonia. Despite organism-sensitive triple antibiotic therapy with linezolid, imipenem and clindamycin from the first day of treatment, progressive abscess formation in multiple skeletal muscles was observed. As a result, repeated surgical interventions became necessary. Due to progressive soft tissue infection, the anti-microbial therapy was changed to a combination of clindamycin and daptomycin. Continued surgical and antimicrobial therapy finally led to a stabilisation of the patients' condition. The clinical course of our patient underlines the existence of a "*PVL-syndrome*" which is independent of *in vitro Staphylococcus aureus *susceptibility. The PVL-syndrome should not only be considered in patients with soft tissue or bone infection, but also in patients with pneumonia. Such a condition, which may easily be mistaken for uncomplicated pneumonia, should be treated early, aggressively and over a long period of time in order to avoid relapsing infection.

## Introduction

Panton Valentine Leukocidin (PVL) positive s*taphylococcal *infection typically presents as life-threateninginfection of soft-tissues and bones [1,2]. PVL-positive s*taphylococcal *infection may also lead to necrotizing pneumonia, a condition which can even be observed before the onset of soft-tissue or bone infection [3,4]. The underlying molecular mechanisms in regard to the progression of PVL-positive *staphylococcal *necrotizing pneumonia and respective methods of bacterial invasion have recently been elucidated [5,6]. The genes encoding the exotoxin PVL are typically present in community-acquired methicillin-sensitive *S. aureus *(CA-MSSA) [3], with about 2–5% of PVL-positive MSSA strains. Nevertheless, this varies markedly in different geographic locations. However, first cases of community-acquired methicillin-resistant *S. aureus *(CA-MRSA) have been reported. Clonal spread of PVL-positive strains and horizontal bacteriophage-dependent PVL-gene transfer thus contribute to an emerging health care problem [7,8]. Although the value of routine PVL testing in staphylococcal infection is currently unclear, both patient and household members should undergo PVL testing in severe or recurrent *S. aureus *infection in order to prevent the spreading of these strains. De-colonization of the patient and respective household members should then be achieved [9,10].

## Case Presentation

We report the case of a 51-year old previously healthy immunocompetent Caucasian male who developed acute illness with fever, dyspnoea and expectoration of bloody sputum during a diving holiday in Croatia. The patient was admitted to a local hospital where empiric antibiotic therapy with azithromycin and ciprofloxacin for radiologically confirmed pneumonia was initiated. Before onset of the acute illness, symptoms suggesting previous (e.g. viral) respiratory tract infection were not observed. After 3 days, the patient was referred to our clinic via an ambulance air service.

**Figure 1:**
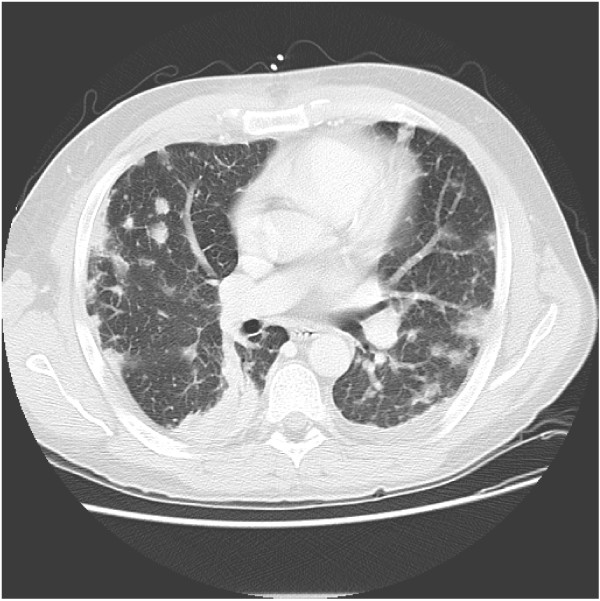
High-resolution (HR-)CT-scan at admission showing signs of severe pulmonary infiltration.

**Figure 2:**
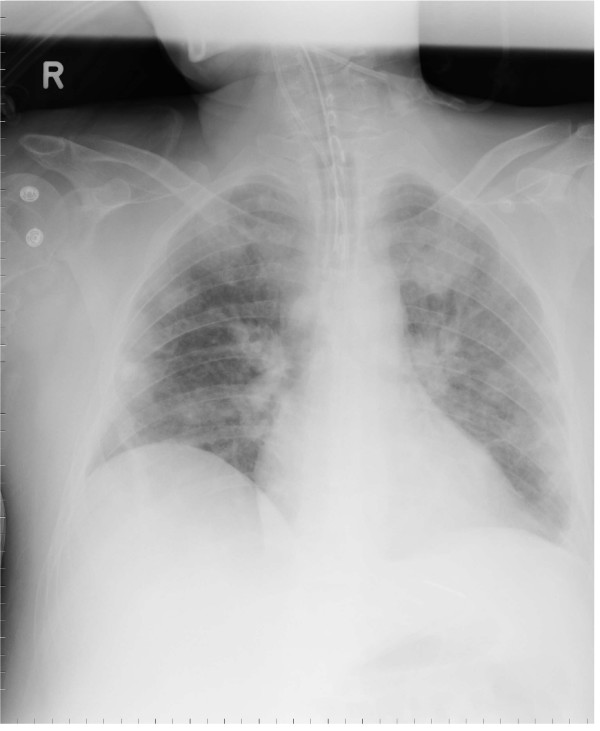
Chest X-ray imaging at admission.

**Figure 3:**
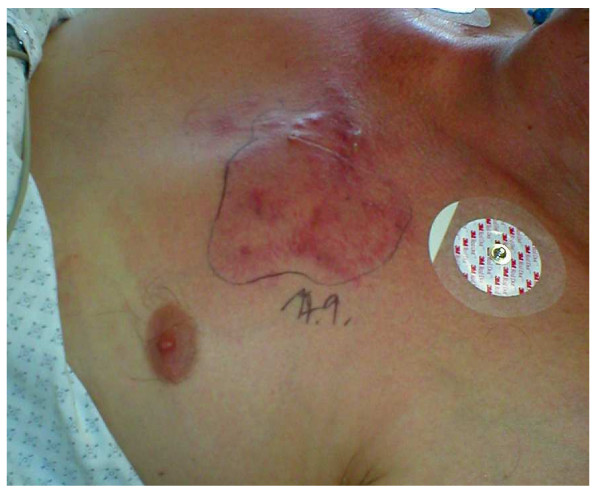
Clinical presentation of progressive furunculosis next to the left sternoclavicular joint.

Upon initial examination, the patient presented with severe sepsis including renal failure. Staphylococcal necrotizing pneumonia was diagnosed based on culture results (blood and broncho-alveolar lavage specimens), clinical criteria, and a high-resolution CT-scan demonstrating signs of severe pulmonary infiltration (Fig. 1, and Chest-X-ray: Fig. 2). Extensive lung tissue necrosis was observed. The antibiotic regimen was changed to a combination of linezolid, imipenem and clindamycin. In correlation to the clinical presentation including high-grade fever [39.2 degrees celsius], laboratory assessment showed signs of severe inflammatory reaction (C-reactive protein 221 [mg/L], procalcitonin 12.4 [μg/L], white blood cell count 15.3 [×10^9^/L], platelet count 182 [×10^9^/L]. Over the ensuing days of treatment, physical examination and radiological imaging revealed abscess formation surrounding the right sternoclavicular joint. Later, progressive diffuse furunculosis next to the left clavicle (Fig. 3) occurred.

Blood cultures and bronchoalveolar samples obtained under triple antibiotic therapy were repeatedly tested positive for methicillin-sensitive *Staphylococcus aureus *(MSSA). Because of the unusual severity of the staphylococcal infection, community-acquired Panton Valentine Leukocidin (PVL)-positive MSSA septic shock was suspected [1]. This was confirmed by PCR demonstration of the respective PVL-genes, as previously reported by us [11]. Furthermore, the isolates were subjected to surface protein A (spa) sequence typing [12]. All isolates investigated exhibited *spa*-type 284 which corresponds to multilocus sequence type ST121. This clonal lineage has been associated with furunculosis in the past decade in Europe and is associated to recent outbreaks [9].

Within 6 days of triple antibiotic therapy with linezolid, imipenem and clindamycin, the patient developed progressive respiratory and haemodynamic failure including development of the *Acute Respiratory Distress Syndrome *(ARDS) requiring mechanical ventilation (FiO_2 _0.8, paO2 79 mmHg, PEEP 15 cmH_2_O, peak inspiratory pressure 33 cmH_2_O). Furthermore, vasopressor support was necessary for a subsequent period of more than two weeks. Ongoing abscess formation in multiple skeletal muscles (Mm. supraspinatus, triceps, quadriceps, gluteus maximus) required repeated surgical interventions and drainage despite organism-sensitive antibiotic therapy from the first treatment day (minimal inhibitory concentrations (mg/L): oxacillin and clindamycin both ≤ 0.25, linezolid ≤ 0.5). Several transesophageal echocardiograms never showed signs of infectious endocarditis. Assessment of an underlying immune disorder showed normal fasting serum blood glucose levels (before onset of severe sepsis), normal quantitative complement levels (C3, C4), and normal immunoglobulin (Ig) levels, including Ig-subclasses. Furthermore, flow assorted cell sorting (FACS) analyses were performed. These analyses revealed the presence of panlymphopenia during sepsis with a normal CD4/CD8 ratio. B- and T-cell numbers were found to be within the limits of normal after reconvalescence.

After two weeks of ongoing metastatic soft tissue infiltration under triple antibiotic therapy (linezolid, imipenem, clindamycin), the anti-microbial therapy was changed to a combination of daptomycin and clindamycin. Continued antimicrobial and surgical therapy finally led to a stabilisation of the patients' condition. The patient was discharged from hospital on day 40. Oral antibiotic treatment with moxifloxacin was prescribed for an additional 45 days. Twelve months after discharge, the patient was found to be symptom-free and to be able to perform routine activities of daily life. PVL testing of the patient's household members revealed no PVL colonization of the patient's family.

## Discussion

The case presented here shows features different from "common" PVL-positive *S. aureus *infection. First, previous case series have reported a high incidence of preceding viral respiratory tract illness [3,6]. In our patient, respective symptoms were not present. Furthermore, we are unaware of any previous report indicating that progression of the disease to septic multiple organ failure including *ARDS*, acute renal and haemodynamic failure occurred under triple organism-sensitive antibiotic therapy with linezolid, imipenem and clindamycin. Interestingly, although this may certainly have been a coincidence, the clinical break-through seemed to be related to a change in the anti-microbial regime. The change to daptomycin and clindamycin was instituted as at this point in time therapy-refractory progressive metastatic soft tissue infection occurred despite organism-sensitive triple antibiotic therapy. Although daptomycin may be considered in cases of severe metastatic soft tissue infections, it seems important to mention that it should not primarily be used for the treatment of pneumonia. This is due to the fact that daptomycin is inactived by pulmonary surfactant [13]. The use of a protein synthesis inhibitor, such as linezolid or clindamycin, may especially be beneficial in the setting of a toxin-mediated staphylococcal infection, and clinical data at least partially support such an approach [14].

The clinical course of our patient may give further evidence that a clinical presentation that is consistent with a "*PVL-syndrome*" might be most appropriate to characterise this life-threatening condition [15]. It should be emphasized, however, that the clinical efficacy of the antimicrobial therapy may be insufficient despite favourable *in vitro *sensitivity testing of the PVL-positive *S. aureus*. This may result in major problems with the management of the disease. Contrary to recently proposed therapeutic principles that aim at shortening the duration of antibiotic therapy whenever possible, this condition should be treated aggressively and over a long period of time in order to avoid relapsing infection. The "*PVL-syndrome*" might create a growing threat in the years to come – physicians treating staphylococcal infection should be watchful and alert.

## Conclusion

In cases of therapy-refractory PVL-positive staphylococcal soft tissue infection, a change in the combination of the anti-microbial therapy may be considered despite favourable *in vitro *sensitivity testing of the *S. aureus*. Daptomycin might be considered in such therapy-refractory cases.

## Competing interest statement

The author(s) declare that they have no competing interests.

## Authors' contributions

JCS, FE, and DH collected all data and drafted the manuscript. DH and WW performed the PCR demonstration of the respective PVL-genes and subjected the respective isolates to *spa*-sequence typing. CS, AK, and AJ coordinated the input of all authors and helped to draft the manuscript. All authors read and approved the final version of the manuscript.

## Consent

The authors declare that they have obtained written informed consent from the patient for publication of this case report.

## References

[B1] Panton PN Valentine FCO Staphylococcal toxin Lancet 1932 1 506 508 10.1016/S0140-6736(01)24468-7

[B2] Moumile K Cadilhac C Lina G Berche P Glorion C Ferroni A Severe osteoarticular infection associated with Panton-Valentine leukocidin-producing Staphylococcus aureus Diagn Microbiol Infect Dis 2006 56 1 95 97 10.1016/j.diagmicrobio.2006.03.007 16678376

[B3] Gillet Y Issartel B Vanhems P Fournet JC Lina G Bes M Vandenesh F Piemont Y Brousse N Floret D Etienne J Association between Staphylococcus aureus strains carrying gene for Panton-Valentine leukocidin and highly lethal necrotizing pneumonia in young immunocompetent patients Lancet 2002 359 753 759 10.1016/S0140-6736(02)07877-7 11888586

[B4] Lina G Piemont Y Godailt-Gamot F Bes M Peter MO Gauduchon V Vandenesch F Etienne J Involvement of Panton-Valentin leukocidin-producing Staphylococcus aureus in primary skin infections and pneumonia Clin Infect Dis 1999 29 1128 1132 10.1086/313461 10524952

[B5] Labandeira-Rey M Couzon F Boisset S Brown EL Bes M Benito Y Barbu EM Vazquez V Hook M Etienne J Vandenesch F Bowden MG Staphylococcus aureus Panton Valentine Leukocidin Causes Necrotizing Pneumonia Science 2007 18 315 1130 1133 10.1126/science.1137165 17234914

[B6] Foster TJ Immune evasion by staphylococci Nat Rev Microbiol 2005 3 12 948 958 10.1038/nrmicro1289 16322743

[B7] Chambers HF Community-associated MRSA-resistance and virulence converge N Engl J Med 2005 352 14 1485 1487 10.1056/NEJMe058023 15814886

[B8] Boyle-Vavra S Daum RS Community-acquired methicillin-resistant Staphylococcus aureus: the role of Panton-Valentine leukocidin Lab Invest 2007 87 1 3 9 10.1038/labinvest.3700501 17146447

[B9] Wiese-Posselt M Heuck D Draeger A Mielke M Witte W Ammon A Hamouda O Successful termination of a furunculosis outbreak due to *luk *S-*luk*F positive, methicillin-sensitive Staphylococcus aureus in a german village by stringent decolonization, 2002–2005 Clin Infect Dis 2007 in press 17479931 10.1086/517503

[B10] Osterlund A Kahlmeter G Bieber L Runehagen A Breider JM Intrafamilial spread of highly virulent staphylococcus aureus strains carrying the gene for Panton-Valentine leukocidin Scand J Infect Dis 2002 34 10 763 764 10.1080/00365540260348554 12477329

[B11] Witte W Braulke C Cuny C Strommenger B Werner G Heuck D Jappe U Wendt C Linde HJ Harmsen D Emergence of methicillin-resistant *Staphylococcus aureus *with Panton-Valentine leukocidin genes in central Europe Eur J Clin Microbiol Infect Dis 2005 24 2 1 5 10.1007/s10096-004-1262-x 15599784

[B12] Harmsen D Claus H Witte W Rothganger J Claus H Turnwald D Vogel U Typing of methicillin-resistant *Staphylococcus aureus *in a university hospital setting by using novel software for spa repeat determination and database management J Clin Microbiol 2003 41 12 5442 5448 10.1128/JCM.41.12.5442-5448.2003 14662923 PMC309029

[B13] Silverman JA Mortin LI Vanpraagh AD Li T Alder J Inhibition of daptomycin by pulmonary surfactant: in vitro modeling and clinical impact J Infect Dis 2005 191 12 2149 2152 10.1086/430352 15898002

[B14] Micek ST Alternatives to Vancomycin for the Treatment of Methicillin-Resistant Staphylococcus aureus Infections Clin Infect Dis 2007 45 S184 S190 10.1086/519471 17712745

[B15] Swaminathan A Massasso D Gotis-Graham I Gosbell I Fulminant methicillin-sensitive Staphylococcus aureus infection in a healthy adolescent, highlighting 'Panton-Valentine leucocidin syndrome' Intern Med J 2006 36 11 744 747 10.1111/j.1445-5994.2006.01220.x 17040363

